# Quantification of contrast agent materials using a new image- domain multi material decomposition algorithm based on dual energy CT

**DOI:** 10.1259/bjro.20180008

**Published:** 2019-04-30

**Authors:** Fazel Mirzaei, Reza Faghihi

**Affiliations:** Medical Radiation Engineering Department, School of Mechanical Engineering, Shiraz University, Shiraz, Iran

## Abstract

**Objective:**

Dual-Energy CT (DECT) is an imaging modality in which the objects are scanned by two different energy spectra. Using these two measurements, two type of materials can be separated and density image pairs can be generated as well. Decomposing more than two materials is necessary in both clinical and industrial CT applications.

**Methods:**

In our MMD, barycentric coordinates were chosen using an innovative local clustering method. Local clustering increases precision in the barycentric coordinates assignment by decreasing search domain. Therefore the algorithm can be run in parallel. For optimizing coordinates selection, a fast bi-directional Hausdorff distance measurement is used. To deal with the significant obstacle of noise, we used Doubly Local Wiener Filter Directional Window (DLWFDW) algorithm.

**Results:**

Briefly, the proposed algorithm separates blood and fat ROIs with errors of less than 2 and 9 % respectively on the clinical images. Also, the ability to decompose different materials with different concentrations is evaluated employing the phantom data. The highest accuracy obtained in separating different materials with different concentrations was 93 % (for calcium plaque) and 97.1 % (for iodine contrast agent) respectively. The obtained results discussed in detail in the following results section.

**Conclusion:**

In this study, we propose a new material decomposition algorithm. It improves the MMD work flow by employing tools which are easy to implement. Furthermore, in this study, an effort has been made to turn the MMD algorithm into a semi-automatic algorithm by employing clustering concept in material coordinate’s assignment. The performance of the proposed method is comparable to existing methods from qualitative and quantitative aspects.

**Advances in knowledge:**

All decomposition methods have their own specific problems. Image- domain decomposition also has barriers and problems, including the need for a predetermined table for the separation of different materials with specified coordinates. In the present study, it attempts to solve this problem by using clustering methods and relying on the intervals between different materials in the attenuation domain.

## introduction

The image formation in CT imaging systems is based on the interaction of X-ray photons with different materials.^1^ On the other hand, the contrast mechanism of the CT images is based on material attenuation coefficients.^2^ Although each material has a unique attenuation coefficient, sometimes in common single-energy CT scans, the constituent materials of the object – despite different atomic number and different chemical structure – are viewed as the same material.^3,4^ For example, in the angiographic studies, calcium plaques of the coronary artery and the iodine contrast agents are viewed as the same material.^5,6^ Because of the problems that arise when two or more materials are viewed the same, DECT imaging system comes into play.^7^ In this imaging modality, the objects are scanned at two different energy levels.^8^ Using two scans, it is possible to separate different materials from each other.^9^.^10^.^9^ In 1976, Macovski and Alvarez stated that in medical diagnosis energy range, the attenuation function of different materials can be written as the weighted sum of Compton Scattering effect and Photoelectric effect components of the total interactions in the object.^11^ Progress in recent years has led to simultaneous scans and therefore, the use of DECT imaging systems were possible in various medical and industrial applications.^12^ Luggage screening,^13^ detection of explosives,^14^ Kidney stone characterization,^15,16^ oncologic applications^17^ and Liver fat quantification^18,19^ are some of the most important applications. Different algorithms for material separation task have been proposed in the literatures.^3,12,20^ Each of these methods does the task in different way.^2,21–23^ The important factor that should be noticed is that most of the algorithms proposed so far are capable of separating two materials, while the applications like Liver fat quantification require separating more than two materials.^24,25^ Because of this, different methods have been proposed in both image and raw-data domains for extending the DECT’s decomposition capability to more than two materials. Mendonca et al proposed one of the most important methods in this field, named MMD – Multi Material Decomposition.^26^ MMD algorithm extends the decomposition process to three materials by incorporating mass conservation and volume-preservation at the same time. Here, a new version of this algorithm is proposed.

In our presentation, the barycentric coordinates are chosen automatically using a local clustering algorithm. This clustering method, makes the search space smaller and which leads to a faster decomposition. Moreover, the bi-directional Hausdorff distance has been utilized for the optimization of the barycentric coordinates selection. In addition, since noise is a very important factor affecting the performance of all decomposition methods,^20^ in this study, the noise reduction step is done using the Doubly Local Wiener Filter Directional Window (DLWFDW).^27^

## methods and materials

Mathematically, DECT imaging systems can be thought as a system of linear equations. The number of equations are equal to the number of materials which we want to decompose.^28^ As stated previously, the attenuation function can be written as the weighted sum of the Compton Scattering and Photoelectric component of the total interactions in the medical diagnosis energy range. We call this method the “Macovski Method”. The other way to form the attenuation function is the “Basis Material Method”.^23,29^ In this method, the attenuation function of different materials is rewritten as the weighted sum of the attenuation coefficient of two arbitrary materials as can be seen in equation 1.

$$\mu \left(E\right)=m_{1}\mu_{1}\left(E\right)+m_{2}\mu_{2}\left(E\right)$$

The two arbitrary materials (m_1_ and m_2_) are represented in the above equation. It can be verified that the accuracy of the basis material method is comparable to Macovski method.^30,31^ Therefore, the attenuation coefficient for different materials can be rewritten using the attenuation coefficients of two arbitrary materials with high clinical significance. For instance, the brain tissue attenuation coefficient can be rewritten using blood and fat attenuation coefficients with proper weights (Here, blood and fat are the basis materials).^30^ Therefore, the decomposition process can be done by solving the equations and selecting two arbitrary materials as basis materials (these equations are obtained by scanning the object at two different energies). The selection of basis materials are totally task specific; for example, in,^22^ atomic number and material density were used as basis materials.

But in most of applications, decomposition of more than two materials is needed. A good example is liver fat quantification. In this task, fat, blood, liver tissue and the contrast agent are four materials that should be decomposed.^25^ For extending decomposition capability of DECT to three materials, one further equation should be added to our linear equation system.^20,24^ Accordingly, a lot of methods are devised for this aim, each trying to improve the decomposition performance to more than two materials. From this point of view, the MMD algorithm proposed by Mendonca et al is one of the most important algorithms. In this algorithm, the problem of adding one further equation to the linear equation system is solved by an assumption that organs of the human body can be considered as an ideal-solution. With this assumption, a third constraint is added to the DECT system of equations by incorporating the mass conservation and volume-preservation principles simultaneously. We will discuss the MMD algorithm below.^26^

### MMD algorithm

The mass attenuation coefficient of an organ or tissue can be written as weighted sum of attenuation coefficients of its constituent materials. It must be noted that the concept of material decomposition is equal to calculation of the constituent material’s concentration in a given solution. In order to consider the mass density, instead of mass attenuation coefficient, the linear attenuation coefficient has been used.

$$\mu_{L}\left(E\right)=\sum_{i=1}^{N}\alpha_{i}\mu_{Li}\left(E\right)$$

Where $V_{i}$ is the volume and $\alpha_{i}$ is the volume fraction of the ***i*_th_** component

$$\alpha_{i}=\frac{V_{i}}{\sum_{j=1}^{N}V_{j}}$$

The matrix form of the above equations is as follows (**Ax =** **B**):

$$\left[\begin{array}{ccc} \mu_{L1}\left(E_{1}\right) & \mu_{L2}\left(E_{1}\right) & \mu_{L3}\left(E_{1}\right)\\ \mu_{L1}\left(E_{2}\right) & \mu_{L2}\left(E_{2}\right) & \mu_{L3}\left(E_{3}\right)\\ 1 & 1 & 1 \end{array}\right]\left[\begin{array}{c} \alpha_{1}\\ \alpha_{2}\\ \alpha_{3} \end{array}\right]=\left[\begin{array}{c} \mu_{L}\left(E_{1}\right)\\ \mu_{L}\left(E_{2}\right)\\ 1 \end{array}\right]$$

The first term of the above equation, **A**,is the attenuation coefficients for materials which needs to be decomposed, the second term, **x**, is the volume fractions of materials, and the term shown by **B** is the pixel data for high and low energy scans. The third row of this matrix equation demonstrates the non-negativity constraint ($\sum_{i=1}^{N}\alpha_{i}=1$) for volume fractions. Therefore, just the volume fractions that satisfy this constraint would be accepted as true response for the material decomposition process.

**Figure 1.**
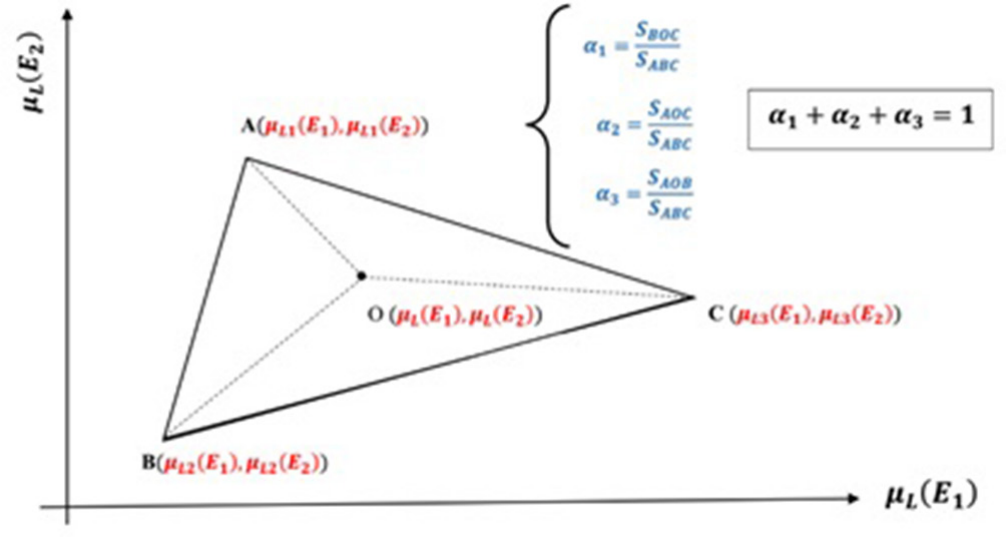
Mathematical interpretation of barycentric coordinate concept for point O inside the triangle. The only volume fraction coefficients are accepted which satisfy non-negativity constraint.

The matrix equation is solved for each pixel of the two input images (high and low energy scans) simultaneously, then the volume fraction of each material in each pixel, is calculated. From geometrical point of view, solving the matrix formula in order to find the volume fractions can be interpreted as finding the barycentric coordinates for a point in the space of linear attenuation coefficients. In this interpretation, the volume fraction of each component is defined as the triangle’s area fraction formed by material attenuation coefficients and inner point (Figure 1). The barycentric coordinates for a point ‘O’ inside the triangle (related to an arbitrary pixel in high and low energy images) and the area fractions formed inside the triangle area and the respective volume fractions can be seen in the Figure 1.

Obviously, it is important for the material decomposition task that the matrix of attenuation coefficients (related to the materials) in (Eq.4) be defined properly.

### Proposed MMD algorithm

#### Local clustering in attenuation domain

**Figure 2.**
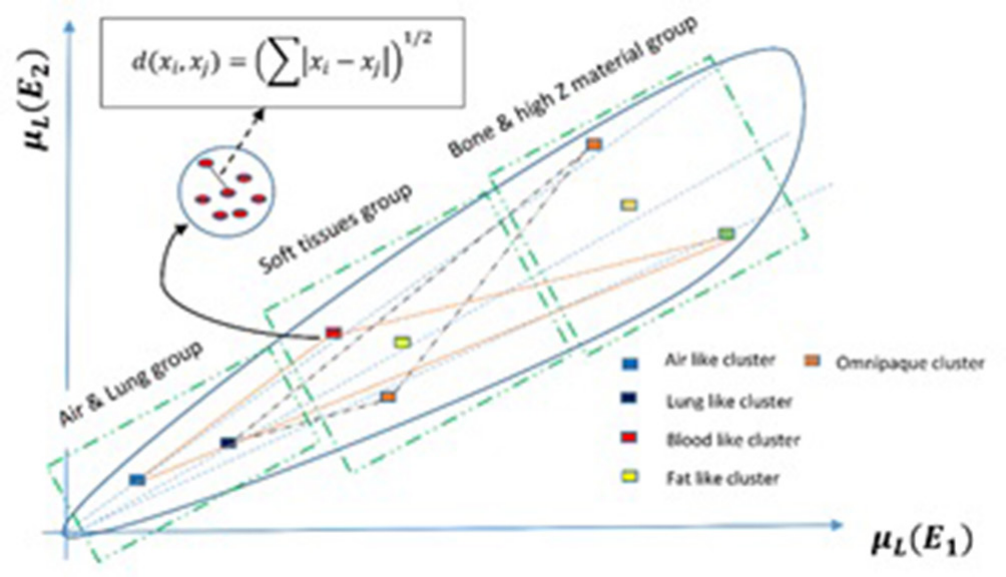
Local clustering in attenuation space. The Euclidean distance between the material point and cluster agent determines whether to put the material in a cluster or not.

As previously mentioned, selection of basis materials plays an important role in the material separation process. On the other hand, choosing material coordinates manually or via look up tables, leads to some biasing errors. Therefore, in this study to obtain higher precision and making semi-automatic (or fully- automatic) decomposition platform, a local clustering algorithm is utilized.^32^ By means of this clustering algorithm, search domain is decreased and separation process done more accurately. For this purpose, a two-dimensional histogram has been created in the linear attenuation domain (Figure 2).

The groups and sub groups in the two-dimensional histogram are chosen according to how different human body constituent materials behave to X-ray photons in certain energies. Several clusters of materials are created in each of these groups, each of which consisting of materials with approximately similar response to X-ray photons. The Euclidean distance between the material point and cluster agent determines whether to put the material in a cluster or not.^33^ So, by calculating the distance between each material point in the 2D histogram and each cluster agent and checking the satisfaction of the pre-determined constraint, all of the data points are clustered. As shown in the Figure 2, decomposition is started by selection of a group of the pre-determined materials. It should be noted that the proposed clustering method is able to update automatically in the situations where the sub groups do not suffice to find the right cluster for each data point; therefore, the sub groups are changed and new coordinates are created. The other important option of the proposed algorithm is its ability to run in parallel; so in this way, several materials can be decomposed at the same time.

Now, the question is: if point O (Figure 1) is not inside the triangle, how will the decomposition process continue? In other words, so far, the linear equation system for points which lie inside the triangle is solved. How can the material decomposition process continue in such situation that point O is not inside? The answer is discussed below.

### Optimization of barycentric coordinates

If the point O was not inside the triangle, the selected coordinates should be modified in such a way that the given point is placed within the new triangle. So the decomposition process continues. Obviously, how to change these coordinates and forming new triangle is the important question in the decomposition procedure. So, this question needs to be answered properly. In the first time of the algorithm running, the cluster agents are chosen as the vertices of the triangle. Then if the point O is located outside the triangle, the vertices are changed and create a new triangle such that the new triangle contains point O. Clearly, the number of triangles that can be selected for doing this (containing point O) is out of count. The criteria which we consider for selecting this new triangle, is the similarity between new triangle and the first one. To do this task, the barycentric coordinates should be optimized. In this section the bi-directional Hausdorff distance measurement method is used to optimize theses coordinates and form the most similar triangle. This method enables the algorithm to choose the most proper triplet group of materials (*e.g.* triangles) among all of the groups.

#### Hausdorff distance measurement

Hausdorff distance measurement is a method for measuring the similarity between any two data sets and has found vast applications in object matching.^34^ This measure can be calculated either one or two-sided. Measuring the similarity between two datasets is usually done by calculating the one-sided Hausdorff distance. This model leads to feasible solutions where the data points are fixed.^35^

Mathematically:

$$d_{H}\left(A,B\right)={min}_{a\in A}{min}_{b\epsilon B}∥a-b∥$$

In the Hausdorff distance formula, “a” and “b” are two arbitrary data points belonging to two arbitrary data sets “A” and “B” respectively. The Hausdorff distance is then defined as the minimum Euclidean distance between “a” and “b”. However, in the decomposition task, these points can be changed from one study to another. Roughly speaking, the coordinates (materials) which are selected automatically or through look up table, can be changed depending on applications and study goals.^36^ So, in this study, the bi-directional (two-sided) Hausdorff distance is used instead of one-side. The bi-directional Hausdorff distance is characterized by the following formula:

$$d_{H}\left(A,B\right)=min\left(d_{H}\left(A,B\right),d_{H}\left(B,A\right)\right)$$

This equation is rewritten to deal with our application in equation 7.

$$d=d_{Hausdorff}\left(\mu_{L},∆\right)={min}_{m\in ∆}\vee \left|m-\mu_{L}\right|\vee$$

In eq.7, $\mu_{L}$ and m are represent vertices of the new and the first triangles. The outline of the Hausdorff distance calculation between two arbitrary sets of “A” and “B” is shown in the Table 1.

**Table 1.** The pseudo code of the Hausdorff distance calculation between two arbitrary sets of “A” and “B”.

***Input*: *Two arbitrary set points A, B***
*d_max_ ← 0*
*E ← A\(A∩B*)
*E_r_ ← random (E*)
*B_r_ ← random (B*)
***for*** *x ϵ E_r_**do:***
*d_min_ ←* ***∞***
***for*** *y ϵ Br **do**:*
*d ← ||x, y||*
***if*** *d <* *d_max_*
***break***
***end***
***if*** *d <* *d_min_*
*d_min_ ← d*
***end***
***end***
***if*** *d_min_ >d_max_*
*d_max_ ← d_min_*
***end***
***end***
***return d_max_***
***Output***: ***Hausdorff Distance***

#### Image denoising in wavelet domain

Noise in the images comes from different sources, and different algorithms have been introduced to eliminate (or reduce) its effect. Since in DECT, X-ray sources have broad energy spectra which overlap each other, noise becomes a significant factor.^37^ In both projection based and image-based approaches, noise is a significant obstacle in the material decomposition process.^38^ Accordingly, the reconstructed images have low SNR and for obtaining good image quality, the patient dose must be increased.^20^ To cope with this problem, in the proposed method “Doubly Local Wiener Filter Directional Window” algorithm was used. This algorithm is first introduced by Peng-Lang Shui in 2005.^27^ The DLWFDW is the generalized version of the “Local Wiener filter” which uses a “Directional Window” for better estimation of the noise.

#### Proposed algorithm

**Figure 3.**
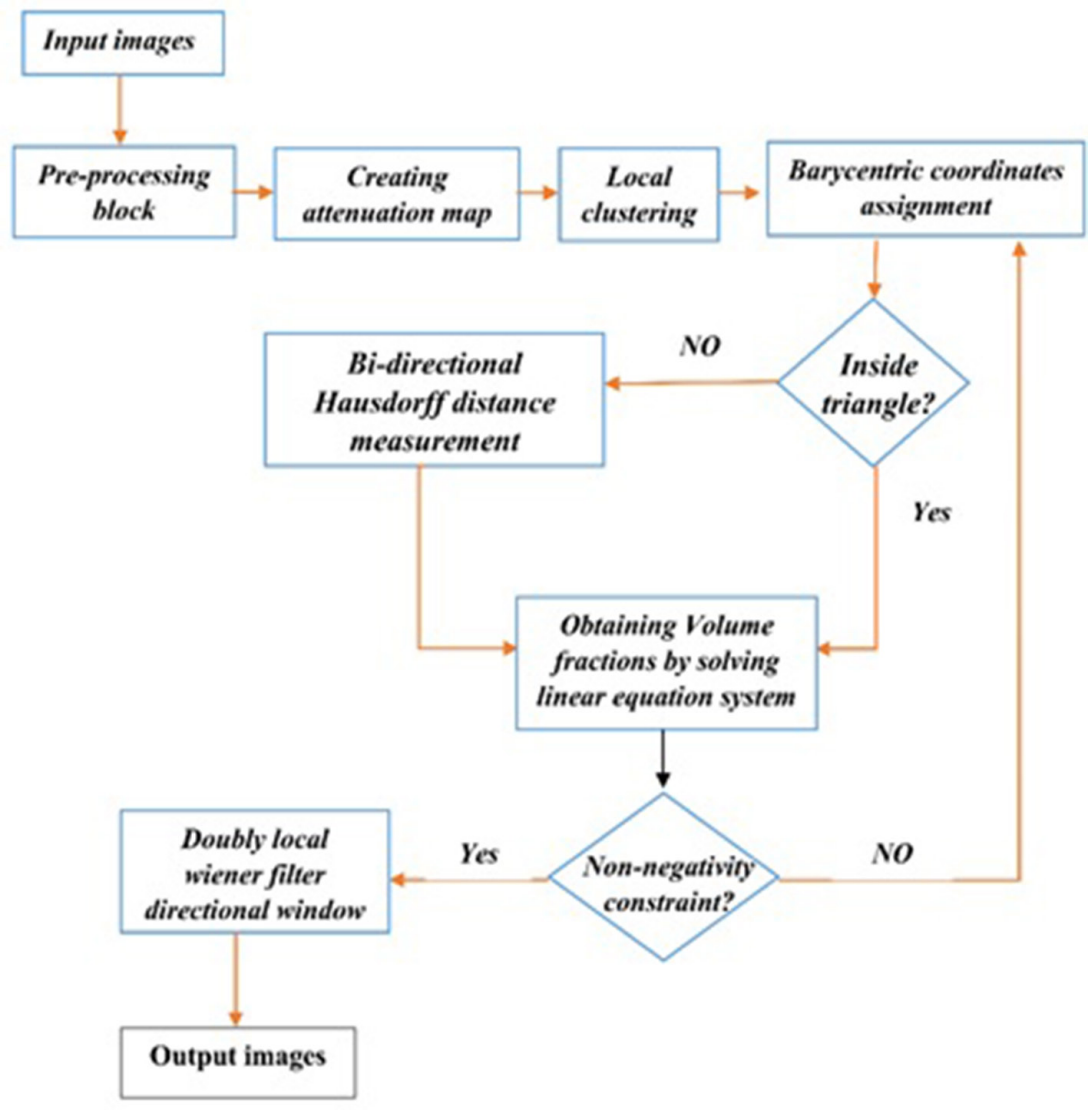
The diagram of proposed algorithm.

The following diagram illustrates the proposed algorithm(Figure 3).

## results

For evaluation of the proposed method, the results are presented in phantom and clinical sections. An angiography study is also designed and different concentration of contrast agent and calcium are decomposed and an image is reconstructed for each of the decomposed materials.

### the clinical results

**Figure 4.**
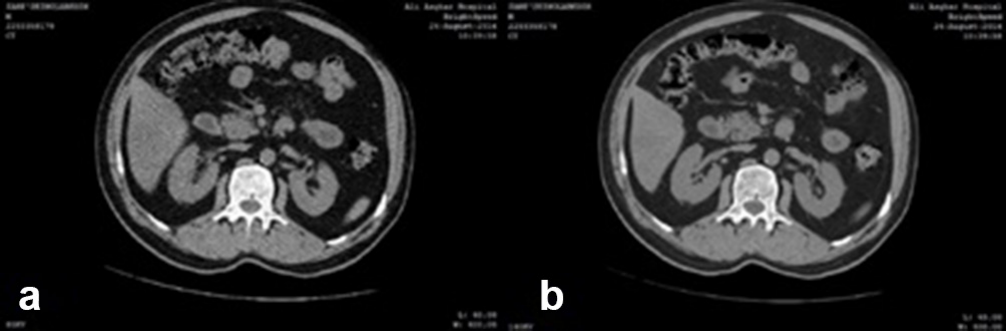
These images show clinical data were taken by GE four slice bright speed scanner. (A) 140, (B) 80 KVP.

**Figure 5.**
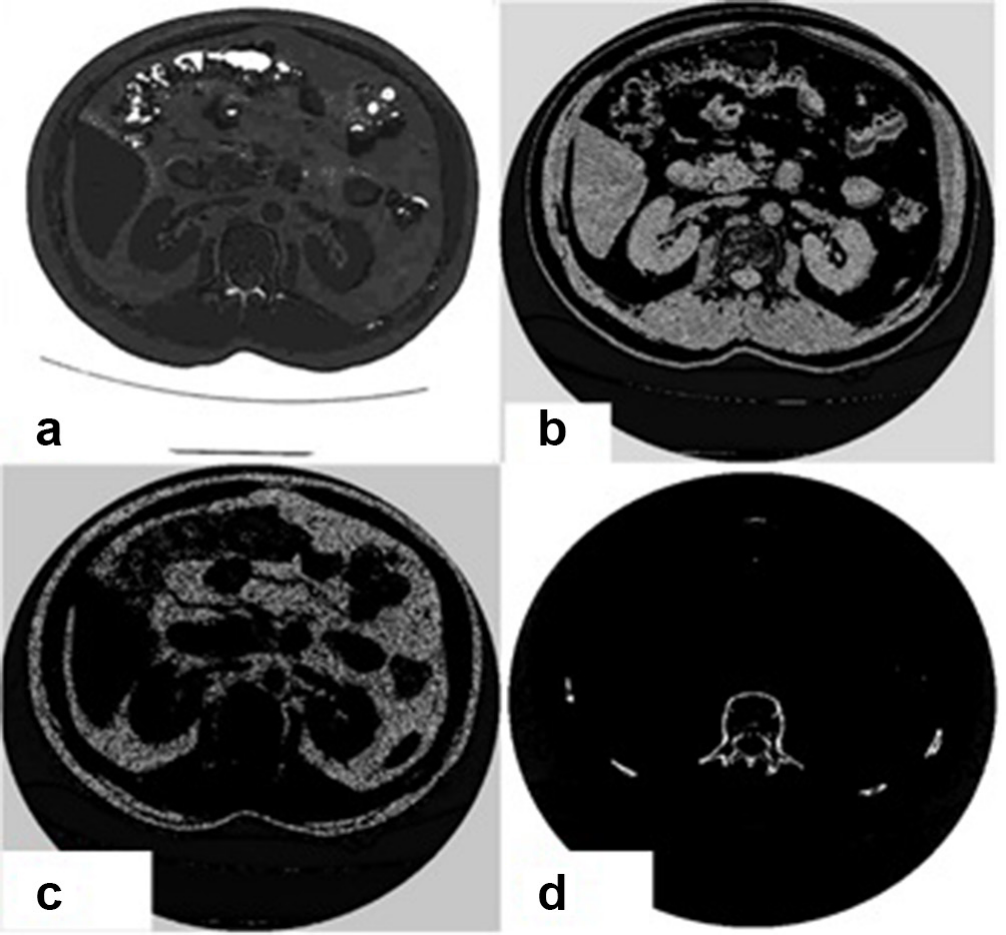
(A) is air fraction, (B) is blood fraction, (C) is fat fraction, and (D) is bone fraction.

The Figure 4 illustrates the 80 and 140 KVP scanned images of the abdominal cross-section. The decomposition process is then applied on these images. Air, Bone, Fat and Blood – as materials of high clinical importance – are chosen as the basis materials. The Figure 5 shows the decomposed material. In this figure the volume fraction images of air, blood, fat and bone are shown respectively. Obviously, the images are of good quality and there is good agreement between the reconstructed images and human body anatomy.

**Figure 6.**
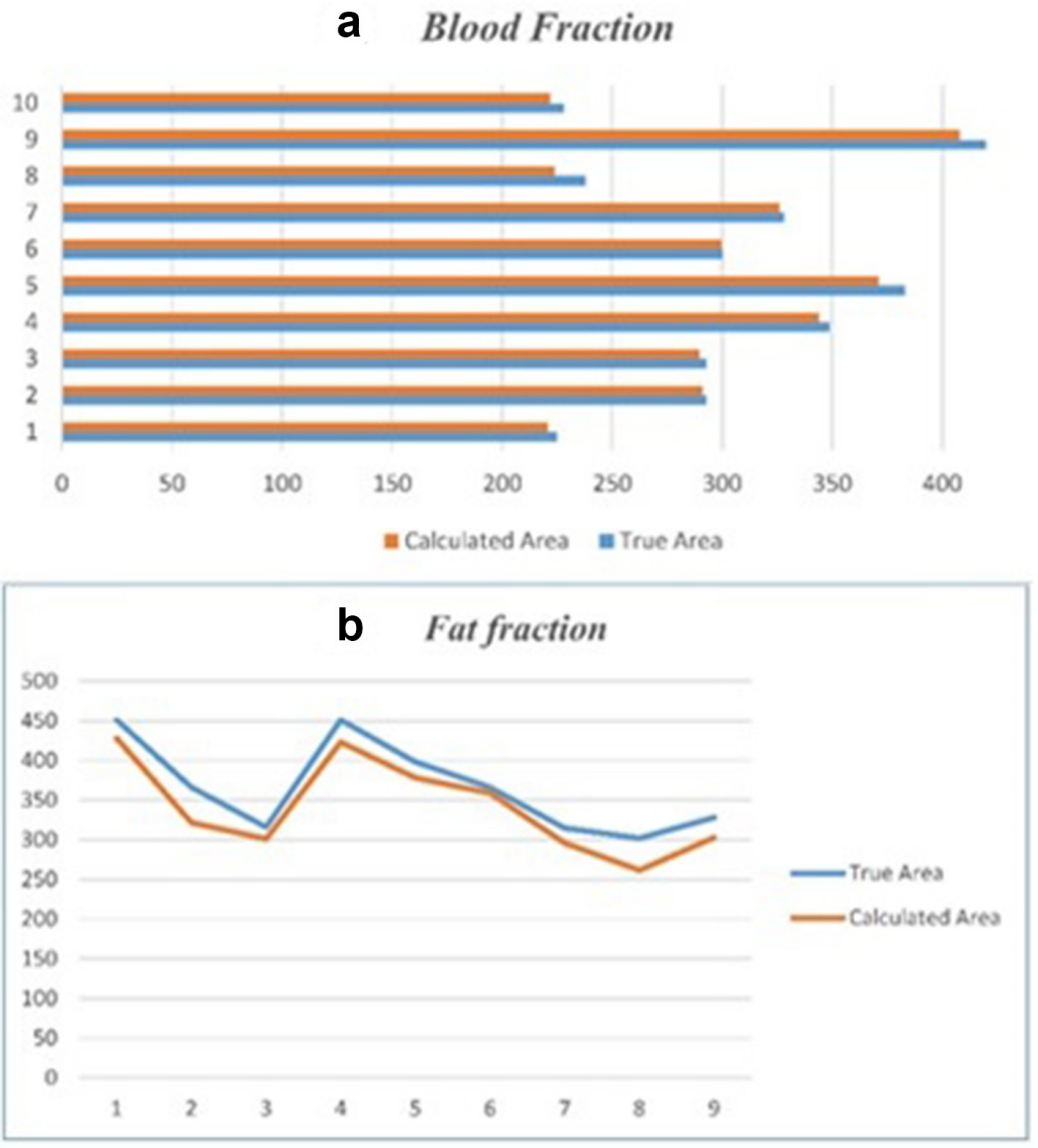
True and calculated area corresponding to Blood ROIs (A) and Fat ROIs (B).

For performance assessment, several regions of interest (ROIs) are selected in each image and in each of them, the ratio of the decomposed to the true area is calculated. The following plots show the results for blood and fat (Figure 6).

### The phantom results: Part 1

**Figure 7.**
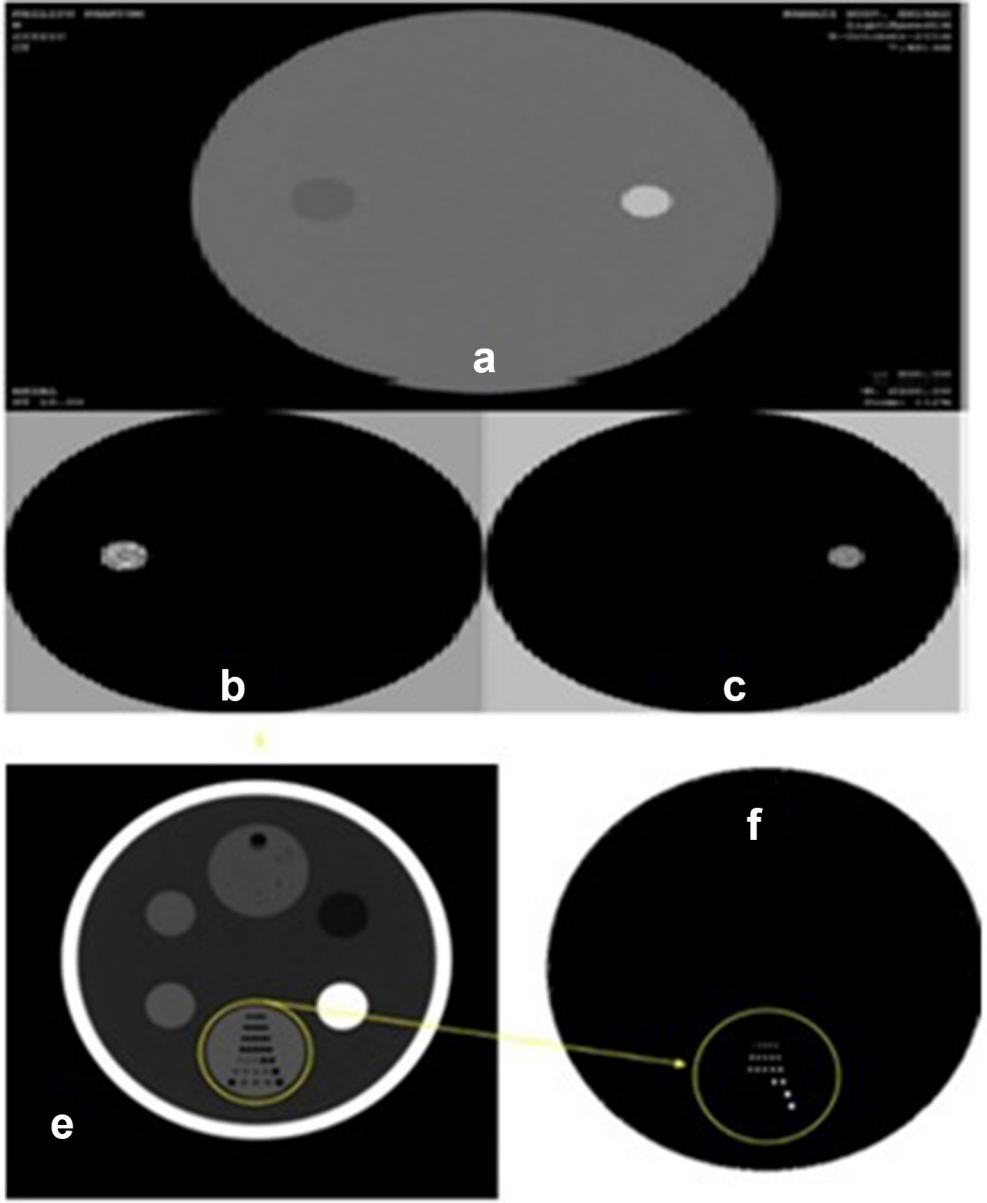
CT quality control phantom’s body section. This section consist of water and Teflon inserts. (A) Original scan, (B) Water component and (C) Teflon component. Head section of CT QA phantom (E) and decomposed Air fractions (F).

In this section, CT quality control phantom is used for studying our proposed method’s performance. This phantom which is used for quality assurance in CT systems, consists of two sections and several materials inserts- *e.g.* Water, Lexan, Acrylic, Teflon and also several air holes in different diameters. This phantom was scanned at 80 and 140 KVP. Then we tried to decompose different materials. Figure 7- (A, B and C) - shows phantom body section with obtained material component images.

As said before, the phantom head section has several pinholes, with different diameters that vary from 1 to 3 mm. The head section of the phantom is used for evaluation of CT images spatial resolution. To evaluate the decomposed image resolution as a criterion for the accuracy of the proposed algorithm, it is attempted to decompose the air-filled pins in the head section. The Figure 7 (E,F) shows the results.

### The phantom results: Part 2

#### Application of angiography

**Figure 8.**
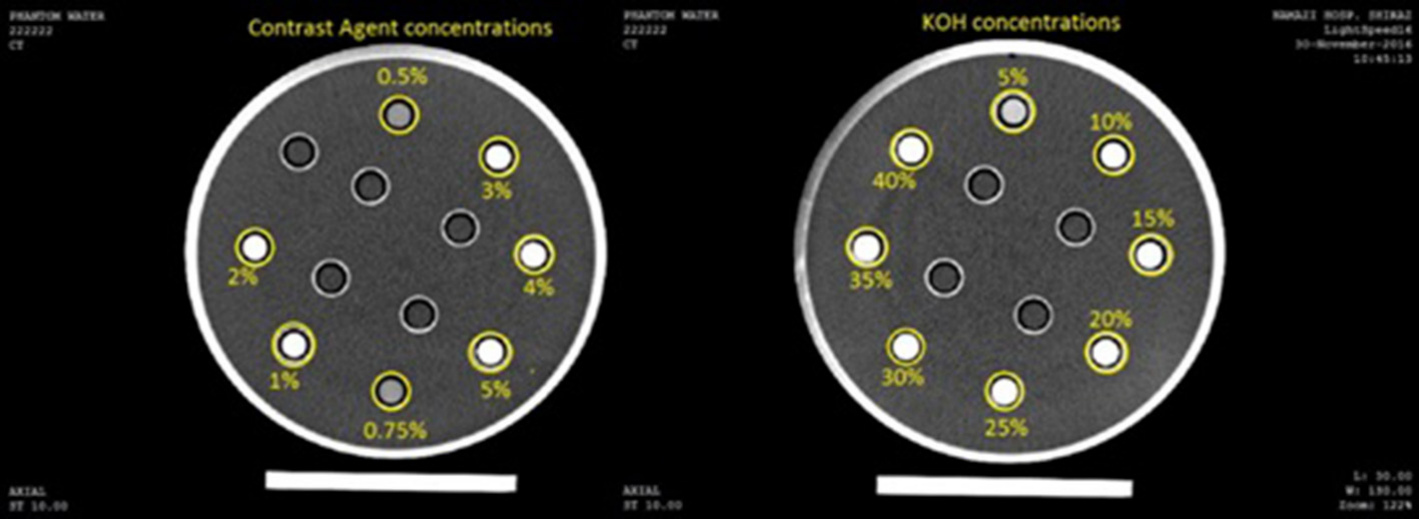
Concentrations and location of vials in the phantom used for the contrast agent and KOH.

In the previous sections, the performance of the proposed method in decomposing different materials was evaluated. In this section, the performance of the algorithm in decomposing different concentration of materials is going to be evaluated. For this purpose, we used a phantom which is designed for angiographic studies. The phantom is designed according to the standards for angiographic and perfusion studies in CT imaging systems and is made of Plexiglas with circular cross-section. It consists of 12 circular vials (each with 12 mm diameter) and each of them contains different material concentration. The rest of the phantom is filled with water. In a typical angiography study, the contrast agent is injected through the veins in certain concentrations. The similar behavior of Calcium plaques in the coronary artery and injected iodine contrast agents has made them hardly distinguishable. To simulate this experiment, vials are filled with certain concentrations of Omnipaque350 contrast agent and KOH – according to Gammaex472 standard – and the phantom is scanned at 80 and 140 KVP (Figure 8).

**Figure 9.**
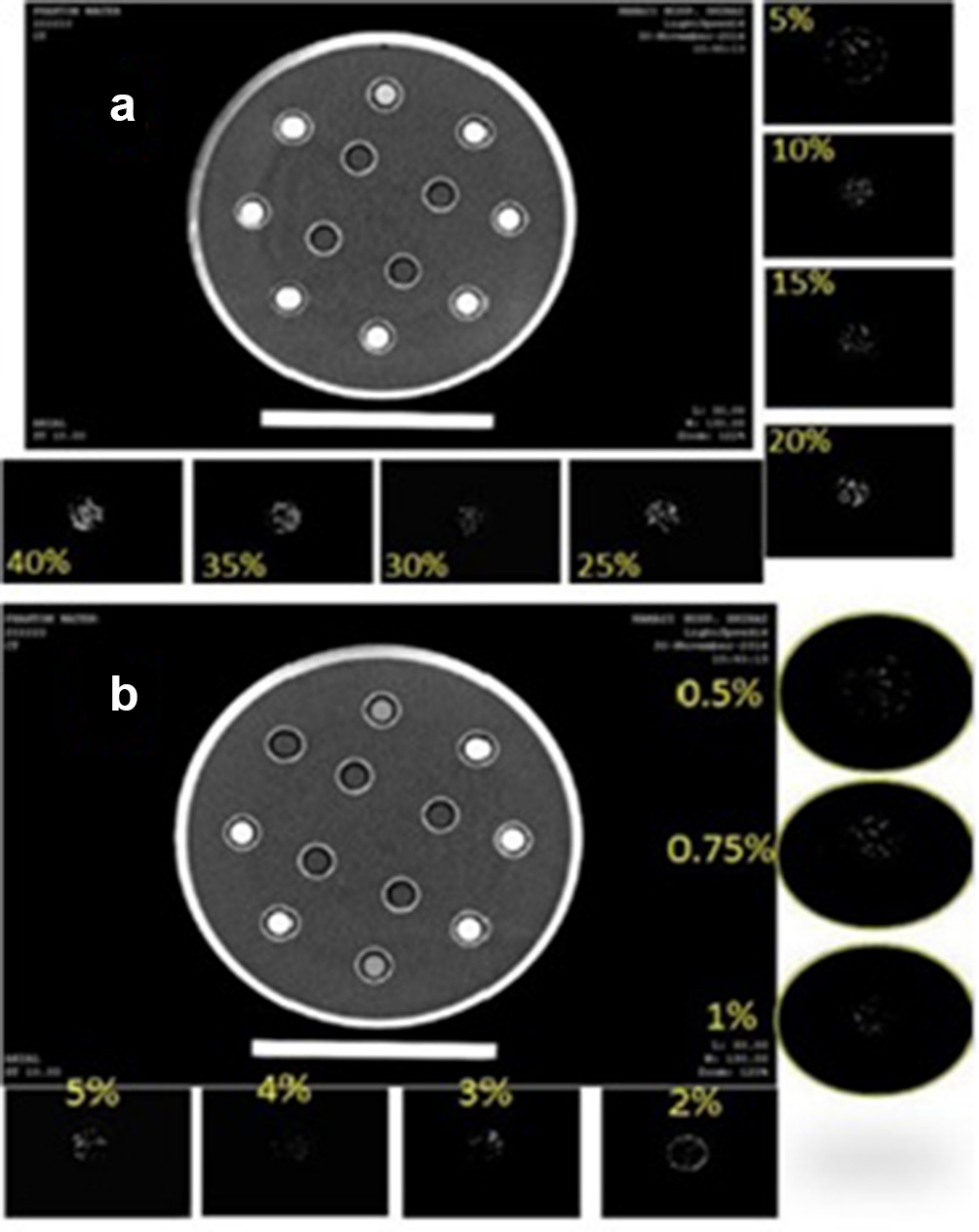
Different concentrations of KOH and the respective image for each concentration (A). Different concentrations of omnipaque 350 and respective images for each concentrations (B).

Then, the algorithm is applied to the images and different concentrations are decomposed. The decomposition results are shown in the following figures (Figure 9).

**Figure 10.**
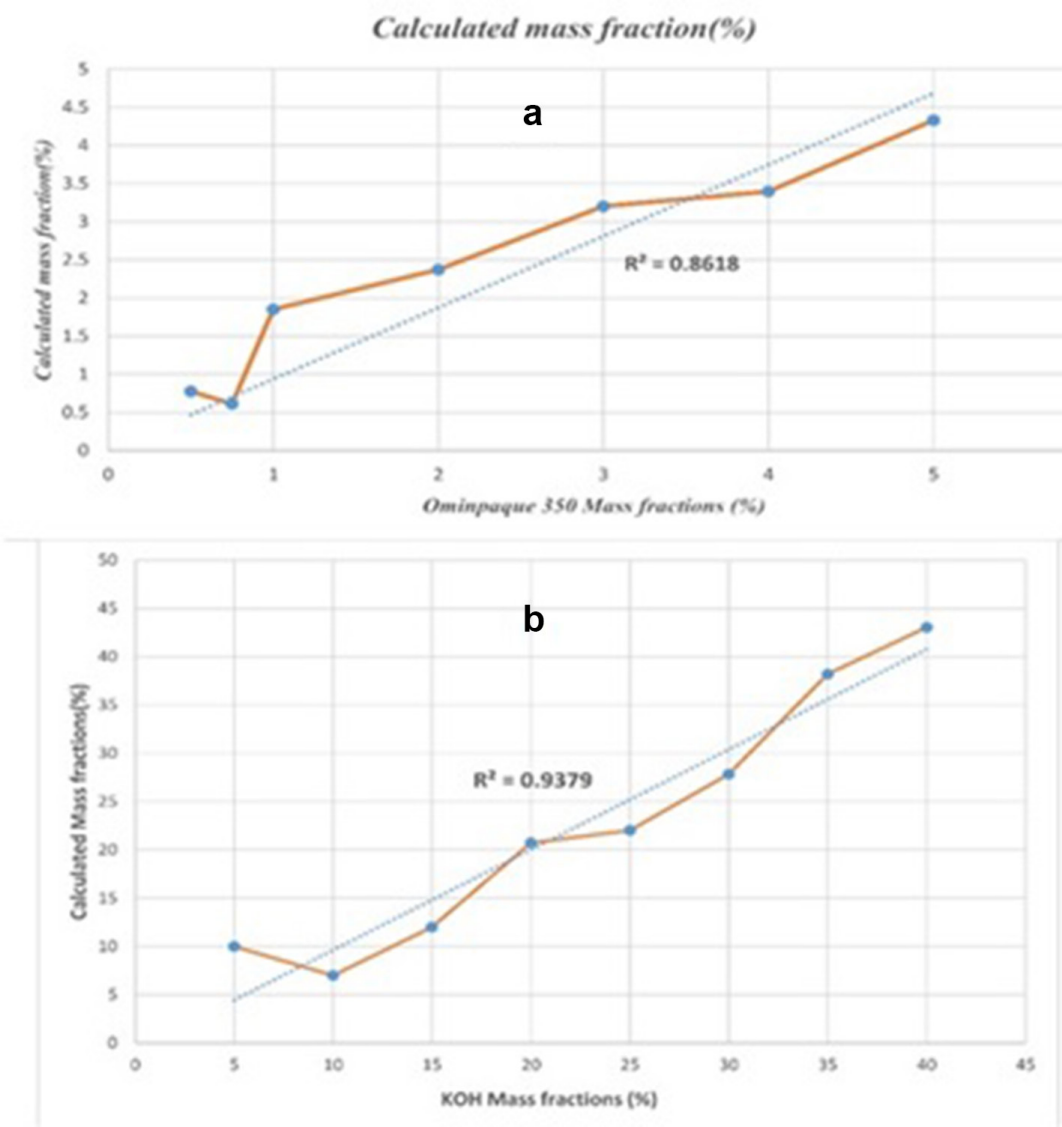
The mass fractions calculated by the proposed algorithm for different concentrations of Omnipaque 350 (A) and KOH (B).

For better evaluation of the proposed algorithm, volume fractions convert into mass fractions and the following graphs will obtain (Figure 10)

## discussion

In this study, a new version of MMD algorithm based on local clustering of Barycentric coordinates was proposed. By means of local clustering, the search domain is decreased which enables us to select Barycentric coordinates (corresponding to material library) automatically. Furthermore, an optimization process based on Hausdorff distance measurement was utilized. Hausdorff distance methods are a general tool for calculating similarity between any two data sets, especially two images. One-sided Hausdorff distance method leads to accurate results when data sets are fixed. For choosing the optimum triangle in the Barycentric domain, bi-directional Hausdorff measurement was used instead of one-sided measurement. The bi-directional Hausdorff method is more practical than the one-sided distance measurement, since, based on study goals, the selected coordinates can change. Moreover, although available algorithms suppress the noise before and after decomposition process, they do not fully consider statistical properties. This problem is addressed by means of DLWFDW. Then, for performance evaluating, the proposed algorithm was applied on clinical images and phantom data. The proposed algorithm separates blood and fat ROIs with errors of less than 2 and 9% respectively. Also, using phantom, the ability to decompose different materials is evaluated and the reconstructed images have a resolution of 1 mm. In order to evaluate the performance of noise reduction step, by adding certain noise values to the images and applying the proposed algorithm to them, noise was reduced by 93 and 89% in the phantom body and phantom head images respectively.

In addition, we designed an experiment to evaluate the performance of the proposed algorithm in separating various concentrations of materials. So, various concentrations of the KOH and the contrast agent were used based on standards. The lowest concentration of KOH and the contrast agent is 5 and 0.5% respectively, which is similar to the actual angiographic concentrations. Also, the lowest reported accuracy in obtaining mass fractions for KOH and contrast agent is 93 and 86%, respectively.

As we stated before, the decomposition process can be done in various manners. Some of this methods perform the decomposition process in projection domain, others in the image domain and the third group perform the task using the combined solutions.

Each of these techniques focuses on separating different materials based on the study goals and also they use various material concentrations, different energy spectra, different detection systems and different phantom materials. These factors together, make the comparison more challenging. In the following, for the general comparison, we listed the recent year’s works and pointed out their features(Table 2).

**Table 2.** General comparison of proposed method with other existing methods

year	Authors	Method description	Material decomposition task	Decomposition Error (%)					
				High Z	Fat	Blood	Iodine	Calcium	HA
2009	Liu et al	Three-material decomposition of mass fraction	Image domain	Up to 18%	Not reported	Not reported	No reported	Up to 26%	Up to 20%
year	Authors	Method description	Material decomposition task	Decomposition Error (%)					
				High Z	Fat	Blood	Iodine	Calcium	HA
2010	H. Q. Le and S. Molloi	A least-squares minimization algorithm was used to decompose materials with the different phantoms study	Image domain, testing on different phantom materials (*e.g.,* PMMA …)	Not reported	Not reported	Not reported	0.0945	Not reported	0.2596
year	Authors	Method description	Material decomposition task	Decomposition Error (%)					
				protein	lipid	blood	iodine	calcium	water
2013	Ding et al	Breast composition measurement with dual energy CBCT	Image Domain	R2 ~ 0.542	R2 ~ 0.962	Not reported	Not reported	No reported	R2 ~ 0.926
year	Authors	Method description	Material decomposition task	Decomposition Error (%)					
				High Z	Fat	blood	iodine	calcium	Polystyrene
2014	Dong, Niu, and Zhu	Combined iterative reconstruction and image-domain decomposition	using total-variation regularization	0.005	Not reported	Not reported	0.07	Not reported	0.061
year	Authors	Method description	Material decomposition task	Decomposition Error (%)					
				High Z	Fat	blood	iodine	CaCl2	water
2015	Li et al	Material Decomposition with a General Volume Constraint for Photon-Counting CT	Image domain	R2 = 0.97	Not reported	Not reported	Not reported	R2 = 0.96	R2 = 0.98
Proposed		Method description	Material decomposition task	Decomposition Error (%)					
				High Z	Fat	blood	iodine	Calcium	Water
		New version of MMD	Image domain	R2 = 0.99	0.09	2.00%	R2 = 0.86	R2 = 0.93	R2 = 0.98

Considering the Table 2, we see high performance of our proposed method. The reason for the undesirable performance of the proposed algorithm in separating Iodine contrast agent is the low concentration of this material which was about 1 mg ml^−1^. If we increase the concentration of the contrast material, the accuracy of the proposed algorithm will increase. For example, if the concentration of the contrast agent were 5 mg ml^−1^, the precision of the decomposition process will reach 97.1%.

As the special case, we compared our method with the MMD algorithm.(Table 3) Maia et.al in^39^ stated that MMD algorithm suffers from a limitation in choosing triangle coordinates. In fact, different energy levels lead to numerical errors and thus triangle coordinates choose incorrectly. In the other words, choosing coordinates manually or via look up tables, leads to bias errors. On this basis, in presented work, we proposed a clustering framework for selecting triangle coordinates. Using that, choosing coordinates is done automatically. In fact, clustering method minimize the search domain and coordinates choose in an optimized way. Also we used bi-directional Hausdorff distance as optimization step. This concept helps the proposed algorithm to avoid bias errors. As stated, Barycentric coordinates in attenuation domain vary from one task to another. So triangles matching is done by means of the Hausdorff concept to avoid numerical errors.

**Table 3.** MMD and proposed method comparison

method	Material selection	Clustering step	Coordinates optimization	de-nosing step	Decomposition accuracy			
					Contrast free	Fat	Contrast enhanced	Blood
Mendonca et al	Manually	None	Look up tables	None	95 %	97%	95%	Not reported
Proposed	Automatically	Yes	Bi-directional Hausdorff distance	DLWFDW algorithm	93%	91%	97.1%	98%

Although Mendonca et.al have suggested that the MMD algorithm has a low sensitivity to noise, Niu et.al^20^ have proven that noise has a significant influence on the performance of decomposition methods in both projection and image domain techniques and also They noted that to eliminate noise effect and also for performing good decomposition and reconstructing high quality images, once have to increase patient dose. Therefore, in order to enhance the performance of the algorithm, as well as eliminating the effects of computational and systematic noise that can disturb the separation process, and also to prevent increasing the patient dose, the proposed method used a de-noising step by means of DLWFDW algorithm.

Considering the fact that the concentration of materials and study goals have a significant impact on the accuracy of the algorithms and each technique focuses on the different situations, the following table compared the MMD with the proposed method.

Considering all the mentioned methods and their characteristics, the proposed algorithm has a satisfactory performance and also it can be improved in future works. On the other hand, it improves the MMD workflow and can be more evaluated by applying it under different conditions.

## Conclusions

We proposed a new version of MMD algorithm which extends the capability of material decomposition process. The results were discussed both qualitatively and quantitatively.
